# Associations between C-reactive protein and individual symptoms of depression in a lower-middle income country

**DOI:** 10.1192/bjo.2024.735

**Published:** 2024-10-03

**Authors:** Elise Fellows, Brett D. M. Jones, John Hodsoll, Nusrat Husain, Ameer B. Khoso, Allan H. Young, Imran B. Chaudhry, M. Ishrat Husain

**Affiliations:** University of Toronto Temerty Faculty of Medicine, Institute of Medical Science, Toronto, Canada; Centre for Addiction and Mental Health, Mood Disorders, Toronto, Canada; Department of Psychiatry, Temerty Faculty of Medicine, University of Toronto, Canada; Campbell Family Mental Health Research Institute, Centre for Addiction and Mental Health, Toronto, Canada; Department of Biostatistics and Health Informatics, Institute of Psychiatry, Psychology and Neuroscience, King's College London, UK; Lancashire & South Cumbria NHS Foundation Trust, London, UK; and Division of Psychology and Mental Health, University of Manchester, UK; Pakistan Institute of Living and Learning, Karachi, Pakistan; Centre for Affective Disorders, Institute of Psychiatry, Psychology, and Neuroscience, King's College London, UK; Division of Neuroscience and Experimental Psychology, School of Biological Sciences, Faculty of Biology, Medicine and Health, University of Manchester, UK; Dow University of Health Sciences, Karachi, Pakistan; and Ziauddin University Hospital, Karachi, Pakistan; Department of Psychiatry, Temerty Faculty of Medicine, University of Toronto, Canada; and Campbell Family Mental Health Research Institute, Centre for Addiction and Mental Health, Toronto, Canada

**Keywords:** Pakistan, major depressive disorder, inflammation, C-reactive protein, lower-middle income country

## Abstract

**Background:**

Data on associations between inflammation and depressive symptoms largely originate from high income population settings, despite the greatest disease burden in major depressive disorder being attributed to populations in lower-middle income countries (LMICs).

**Aims:**

We assessed the prevalence of low-grade inflammation in adults with treatment-resistant depression (TRD) in Pakistan, an LMIC, and investigated associations between peripheral C-reactive protein (CRP) levels and depressive symptoms.

**Method:**

This is a secondary analysis of two randomised controlled trials investigating adjunctive immunomodulatory agents (minocycline and simvastatin) for Pakistani adults with TRD (*n* = 191). Logistic regression models were built to assess the relationship between pre-treatment CRP (≥ or <3 mg/L) and individual depressive symptoms measured using the Hamilton Depression Rating Scale. Descriptive statistics and regression were used to assess treatment response for inflammation-associated symptoms.

**Results:**

High plasma CRP (≥3 mg/L) was detected in 87% (*n* = 146) of participants. Early night insomnia (odds ratio 2.33, 95% CI 1.16–5.25), early morning waking (odds ratio 2.65, 95% CI 1.29–6.38) and psychic anxiety (odds ratio 3.79, 95% CI 1.39–21.7) were positively associated, while gastrointestinal (odds ratio 0.38, 95% CI 0.14–0.86) and general somatic symptoms (odds ratio 0.34, 95% CI 0.14–0.74) were negatively associated with inflammation. Minocycline, but not simvastatin, improved symptoms positively associated with inflammation.

**Conclusions:**

The prevalence of inflammation in this LMIC sample with TRD was higher than that reported in high income countries. Insomnia and anxiety symptoms may represent possible targets for personalised treatment with immunomodulatory agents in people with elevated CRP. These findings require replication in independent clinical samples.

Major depressive disorder (MDD) is a debilitating mental disorder characterised by emotional, cognitive and physical symptoms that impact an individual's ability to function in multiple domains.^1^ The global impact of MDD cannot be understated, with it being one of the leading causes of disability.^2^ As of 2023, the World Health Organization estimates that approximately 5–6% of adults experience MDD around the world.^3^ This figure is expected to grow, as the incidence of MDD appears to be increasing steadily over time.^4^ Making matters worse, traditional pharmacotherapies that act on monoamine neurotransmitter systems such as selective-serotonin-reuptake-inhibitors (SSRIs) and selective noradrenaline reuptake inhibitors (SNRIs)^5^ are often ineffective, of minimal benefit or poorly tolerated. Up to 50%^4^ of people with MDD do not achieve a response with these medications and experience treatment-resistant depression (TRD). This suggests that other mechanisms beyond those directly involving monoamine neurotransmission are likely contributing to the development of depressive symptoms in a high proportion of people. Clearly a deeper understanding of the biology of MDD is urgently needed to facilitate the development of alternative treatments and address the unmet needs of individuals with TRD across populations and settings.

Converging evidence supports the role of dysregulation in the innate immune system in the aetiology of MDD, and elevated inflammation is thought to contribute to treatment resistance.^6^ C-reactive protein (CRP), an acute-phase reactant protein commonly used as a marker of peripheral inflammation, has been reported to be higher in patients with TRD compared with non-TRD patients.^6,7^ Furthermore, higher levels of interleukin-6 (IL-6), a cytokine which induces CRP synthesis in the liver,^8^ have been detected in individuals with MDD whose symptoms were refractory to SSRIs and SNRIs when compared with those who responded to these medications.^5^ While standard antidepressant medications have some anti-inflammatory activity,^9^ they alone may not be adequate to address underlying inflammation in a subset of people with MDD. In theory, adjunctive medications to target inflammation may improve antidepressant treatment efficacy. However, clinical trials of repurposed anti-inflammatory agents in MDD and TRD have reported mixed results.^10^ A recent systematic review and meta-analyses concluded that no clear recommendations could be made regarding the use of anti-inflammatories in MDD given the current state of research in this area.^11^ Inflammation has, however, been linked to individual features of depression, such as anhedonia, appetite dysregulation, sleep disturbances, low energy and suicidality.^12^ It is possible that anti-inflammatory medications may improve these symptoms individually, though research in this area is limited.

Furthermore, despite nearly 40% of the global population residing in lower-middle income countries (LMICs),^13^ most research on MDD, its treatment and pathophysiology, has involved populations in high income countries (HICs). The high burden of mental illness in LMICs, together with limited access to evidence-based mental health interventions,^14^ highlights the need for studies to identify effective and accessible psychiatric treatment protocols in these settings. The goal of this study is to determine the prevalence of inflammation in people with TRD from studies in a LMIC setting, and to explore associations between specific depressive symptoms and peripheral inflammation in this population.

## Method

### Study design

This study is a cross-sectional secondary analysis of data pooled from two multicentre randomised controlled trials (RCTs) in Pakistan, evaluating the safety and efficacy of adjunctive anti-inflammatory medications in adults with TRD. Treatment resistance in both trials was defined as non-response to treatment with two antidepressant medications for a minimum of 4 weeks at their minimum effective dosage during the current major depressive episode. Both trials followed British National Formulary and Maudsley Prescribing Guidelines for determining minimum effective medication dosages. CRP was measured at baseline and after 12 weeks of treatment in both studies.

The first trial was a multicentre, two arm, placebo-controlled RCT investigating simvastatin (20 mg daily) added to standard treatment (i.e. psychotropic medications and out-patient psychiatric care) versus placebo added to standard treatment (ClinicalTrials.gov Identifier: NCT03435744). Participants included 150 out-patients with MDD and TRD, aged 18–75 years treated at psychiatric clinics across five urban centres in Pakistan, with a score of 14 or higher on the 24-item Hamilton Depression Rating Scale (HAMD-24). Exclusion criteria included: diagnosis of psychotic disorder or bipolar disorder; current use of statins; unstable medical or neurological problems; autoimmune or inflammatory disorders; alcohol or substance use disorder; active suicidal ideation; and pregnancy or breastfeeding. This study was approved by The National Bioethics Committee of the Pakistan Health Research Council. Detailed methodology has previously been reported.^15^

The second was a 12-week, multicentre, placebo-controlled RCT of minocycline (200 mg daily) added to standard treatment for TRD versus placebo added to standard treatment (ClinicalTrials.gov identifier: NCT02263872). The study included 41 out-patients aged 18–65 years treated at four out-patient psychiatric clinics in Karachi, Pakistan. Exclusion criteria included: relevant medical illnesses or concomitant penicillin therapy; concomitant anticoagulant therapy; presence of a seizure disorder; current use of valproic acid; diagnosis of substance use disorder; pregnant or breastfeeding; and the presence of a primary psychotic disorder. This study was approved by the ethics committee of the Karachi Medical and Dental College and Dow University of Health Sciences, Pakistan.^16^

Ethics approval and informed consent was not required for the secondary analysis. Both original trials obtained ethics approval and written informed consent from research participants.^15,16^

### Demographic and clinical variables

CRP was selected as the sole biomarker of inflammation in this study because of the availability of data across both clinical trials, as well as its known associations with MDD.^17^ Inflammation was defined as blood CRP levels of greater than or equal to 3 mg/L. This cut-off was chosen to reflect guidelines from the United States Centers for Diseases Control and Prevention, and the American Heart Association, which considers CRP levels over 3 mg/L to be indicative of elevated inflammation and high risk for the development of cardiovascular disease.^18^ This cut-off has previously been used to represent low-grade inflammation in other depression studies.^19,21^

Demographic and clinical variables were included based on their availability in the entire sample across both trials. Demographic variables included age, gender, marriage status (‘single’, ‘married’ and ‘widowed or divorced’), years of education and socioeconomic status (SES) (‘lower’ and ‘middle/upper’). Lower SES was defined as a monthly income of 17 000 Pakistani rupees or lower. Clinical variables included weight in kilograms and body mass index (BMI), use of anticholinergic medication, typical and atypical antipsychotic or benzodiazepine medications, number of hospital admissions and clinical assessment scores. Clinical assessments included the Hamilton Depression Rating Scale (HAMD),^22^ General Anxiety Disorder-7 (GAD-7),^23^ Clinical Global Impression scale (CGI)^24^ and Patient Health Questionnaire-9 (PHQ-9).^25^ Individual depressive symptoms were measured as individual item scores on the first 17 items of the HAMD.

### Statistical analysis

The sample was divided based on inflammatory status into ‘high CRP’ (CRP ≥ 3 mg/L) and ‘non-inflamed’ (CRP < 3 mg/L) groups. Between-group comparisons were performed using Mann–Whitney U Test for continuous and ordinal variables and with Chi-squared or Fisher exact test for categorical variables.

Following a similar statistical strategy applied previously,^21^ a series of logistic regression models were built to assess inflammatory status as a binary dependent variable (CRP ≥ or <3 mg/L), with one symptom, clinical or demographic variable included in each model as an independent variable. Age, gender, socioeconomic status and BMI were selected a priori as covariates due to their known influence on both depression and inflammation. CRP was also examined as a continuous variable using linear regression, with the same independent variables and covariates. The distribution of CRP was positively skewed and thus was normalised with log10 transformation. Statistical significance was defined as *P*-values of <0.05. Results from this exploratory analysis were not corrected for multiple comparisons.

Finally, we explored whether anti-inflammatory treatment improved symptoms associated with inflammation in logistic regression for participants with high CRP. Regression analysis was used for simvastatin treatment, with HAMD item score at 12 weeks as the dependent variable, and treatment group (placebo or simvastatin) and baseline HAMD item score as independent variables, with the covariates included. Response to treatment with minocycline was assessed using descriptive statistics due to the small number of participants from the original trial (*n* = 16) with CRP ≥ 3 mg/L. All statistical analyses were performed with statistical computing software R.^26^

## Results

### Sample and baseline characteristics

The total sample across both trials included 191 adult out-patients with MDD and TRD. Baseline CRP data were available for 171 participants. Of these, three were omitted because of missing weight data, leaving a total of 168 participants in the final sample.

Baseline characteristics and comparisons between high CRP and non-inflamed groups are summarised in Table 1. The final sample for the primary analysis included 92 females (55%) and 75 males (45%) with a mean age of 37.1 (9.6) years. Most participants were married (80%), with an average of 6.3 (5.4) years of education. A total of 60% of participants in the sample fell into the ‘lower’ socioeconomic class, and all participants were prescribed an antidepressant. In all, 87% (*n* = 146) had elevated plasma CRP at baseline.

**Table 1:** Baseline demographic and clinical variables

Demographic variables	Entire group (*n* = 168)	CRP ≥3 (*n* = 146)	CRP < 3 (*n* = 22)	*P*
	Mean (s.d.)	Mean (s.d.)	Mean (s.d.)	
Age	37.1 (9.6)	37.7 (9.3)	32.5 (10.4)	0.014^a^
Gender (*n*)				0.242^b^
Female	92	83	9	
Male	76	63	11	
Marriage status (*n*)				0.900^c^
Single	25	21	4	
Married	134	117	17	
Widowed/Divorced	9	8	1	
Years of education	6.3 (5.4)	6.1 (5.3)	7.7 (5.6)	0.199^a^
Socioeconomic status (*n*)				0.850^b^
Lower	100	86	14	
Middle/Upper	68	60	8	
Clinical variables				
Weight (kg)	68.1 (13.1)	68.5 (13.3)	66.0 (11.3)	0.439^a^
BMI	26.81 (5.2)	27.0 (5.3)	25.5 (4.2)	0.196^a^
Medications (*n*)				
Taking antidepressant	168	146	22	NA
Taking anticholinergic	18	13	5	0.065^c^
Taking antipsychotic (typical)	6	6	0	NA
Taking antipsychotic (atypical)	15	13	2	NA
Taking benzodiazepine	48	38	10	0.104^b^
Number of hospitalisations (*n*)				0.021^a^
0	128	107	21	
1	24	23	1	
2	15	15	0	
3	0	0	0	
4	1	1	0	
HAMD-total	31.0 (7.1)	31.2 (7.4)	29.8 (4.9)	0.633^a^
PHQ-9	16.7 (4.1)	16.7 (4.3)	16.3 (3.8)	0.618^a^
GAD-7	13.1 (3.3)	13.1 (3.2)	13.0 (4.0)	0.839^a^
CGI	4.4 (0.9)	4.4 (0.9)	4.1 (0.8)	0.219^a^

CRP, C-reactive protein; BMI, body mass index (weight in kilograms divided by height in metres squared); HAMD, Hamilton Depression Rating Scale; PHQ-9, Patient Health Questionnaire-9; GAD-7, General Anxiety Disorder-7; CGI, Clinical Global Impressions.

Statistical tests: a. Mann–Whitney U Test.

b. Chi-Squared Test.

c. Fisher's Exact Text.

Units: CRP measured in mg/L.

Demographic and clinical variables that were distributed unequally between high CRP and non-inflamed groups included age (*P* = 0.014) and number of hospitalisations (*P* = 0.021), with values being higher for individuals with high CRP.

### Associations between clinical and demographic variables and high CRP in regression analysis

**Fig. 1:**
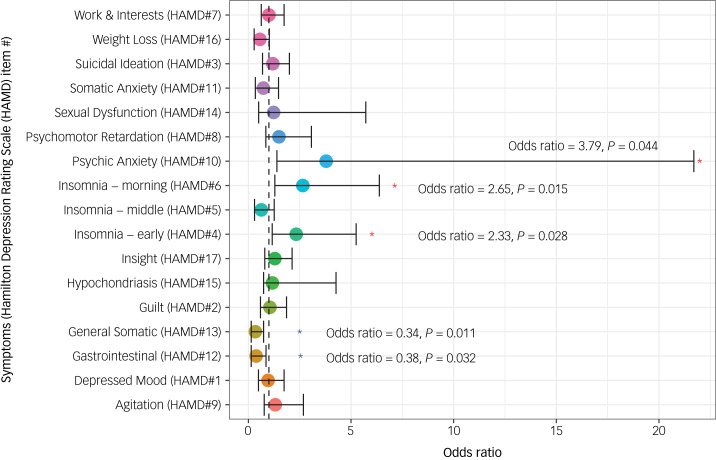
Estimated odds ratios for inflammation (C-reactive protein ≥ 3 miligrams per litre (mg/L).

Results of logistic regression are summarised in Table 2 and Fig. 1. Symptoms that were associated with a higher likelihood of inflammation were: insomnia early in the night (HAMD item#4) (odds ratio 2.33, 95% CI 1.16–5.25, *P* = 0.028), early morning insomnia (HAMD item#6), (odds ratio 2.65, 95% CI 1.29–6.38, *P* = 0.015) and psychic anxiety (HAMD item#10) (odds ratio 3.79, 95% CI 1.39–21.7, *P* = 0.044). Gastrointestinal symptoms and/or low appetite (HAMD item#12) (odds ratio 0.38, 95% CI 0.14–0.86, *P* = 0.032) and general somatic symptoms (HAMD item#13) (odds ratio 0.34, 95% CI 0.14–0.74, *P* = 0.011) were negatively associated with inflammation. The variable ‘number of hospitalisations’ was heavily skewed because of a high number of 0 values (*n* = 128, 76%) and was thus omitted from logistic regression analysis. No other clinical or demographic variables were associated with CRP status in logistic regression. Logistic model plots and ROC curves are provided in Supplementary Figures 1 and 2, respectively.

**Table 2:** Associations between peripheral inflammation and depressive symptoms, clinical and demographic variables in logistic regression

Demographic variables	Odds ratio	95% CI		*P*
		Lower	Upper	
Marriage status				
Single	2.18	0.56	10.4	0.290
Married	0.46	0.09	1.78	0.290
Widowed/Divorced	0.48	0.04	11.3	0.568
Years of education	0.86	0.86	1.06	0.381
Clinical variables				
Weight (kg)	1.02	0.96	1.09	0.499
Taking anticholinergic	2.25	0.62	7.28	0.190
Taking antipsychotic (atypical)	0.73	0.12	3.21	0.727
Taking benzodiazepine	0.70	0.92	6.23	0.074
HAMD-total	1.02	0.96	1.10	0.554
PHQ-9	0.37	0.94	1.18	0.371
GAD-7	0.91	0.86	1.15	0.908
CGI	1.42	0.82	2.53	0.219
Symptom - HAMD item #				
Depressed mood – 1	0.96	0.49	1.74	0.811
Guilt – 2	1.05	0.59	1.86	0.870
Suicidal ideation – 3	1.19	0.69	2.00	0.522
Insomnia (early) – 4	2.33	1.16	5.25	0.028
Insomnia (middle) – 5	0.63	0.30	1.26	0.200
Insomnia (morning) – 6	2.65	1.29	6.38	0.015
Work and activities – 7	1.00	0.63	1.74	0.987
Psychomotor retardation – 8	1.49	0.85	3.07	0.211
Agitation – 9	1.31	0.77	2.68	0.381
Psychic anxiety – 10	3.79	1.39	21.7	0.044
Somatic anxiety – 11	0.73	0.34	1.47	0.407
Gastrointestinal – 12	0.38	0.14	0.86	0.032
General somatic – 13	0.34	0.14	0.74	0.011
Sexual dysfunction – 14	1.23	0.50	5.72	0.716
Hypochondriases – 15	1.17	0.74	4.27	0.227
Weight loss – 16	0.56	0.28	1.02	0.724
Insight – 17	1.29	0.80	2.13	0.305

HAMD, Hamilton Depression Rating Scale; PHQ-9, Patient Health Questionnaire-9; GAD-7, General Anxiety Disorder-7; CGI, Clinical Global Impressions.

Results of linear regression are difficult to interpret and may lack validity due to high variability in plasma CRP in the sample and heteroscedasticity in the residuals and are thus not presented here. A summary of results from linear regression is available in Supplementary Table 1 available at https://doi.org/10.1192/bjo.2024.735.

High variability in CRP may reflect the presence of active infection in some participants at the time of measurement, typically considered plasma CRP > 10 mg/L.^27^ While infection cannot be confirmed, we repeated the logistic and linear regressions while omitting people with baseline plasma CRP > 10 mg/L (*n* = 33) to account for this possibility. In logistic regression, the same symptoms remained significantly associated with CRP status, with results displayed in Supplementary Table 2. Symptom categories positively associated with CRP in linear regression were early waking insomnia (HAMD item#4) (*B* = 0.49, CI 0.15–0.84, *P* = 0.005), work and activities (HAMD item#7) (*B* = 0.36, CI 0.11–0.61, *P* = 0.005), psychomotor retardation (HAMD item#8) (*B* = 0.30, CI 0.04–0.56, *P* = 0.026), agitation (HAMD item#9) (*B* = 0.0.32, CI 0.04–0.59, *P* = 0.023) and psychic anxiety (HAMD item#10) (*B* = 0.0.47, CI 0.21–0.73, *P* ≤ 0.001). Symptoms negatively associated with CRP were general somatic symptoms (HAMD item#13) (*B* = 0.−0.15, CI −0.88 to −0.13, *P* = 0.008) and weight loss (HAMD item#16) (*B* = −0.53, CI −0.84 to −0.22, *P* = 0.001). CGI scores were positively associated with CRP (*B* = 0.38, CI 0.08–0.68, *P* = 0.012). The results of linear regression are summarised in Supplementary Table 3.

### Associations between adjunctive anti-inflammatory treatments and change in symptom scores for symptoms associated with high CRP

**Fig. 2:**
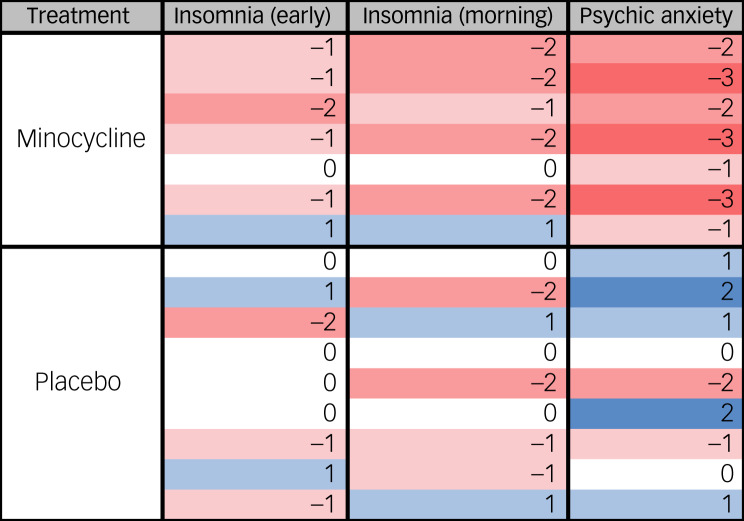
Change in depression symptom scores pre to post-treatment with minocycline versus placebo for symptoms associated with high plasma CRP (≥3 mg/L)^33^. Heat map of change in HAMD item score for insomnia (early) (HAMD#4); insomnia (morning) (HAMD#6); and psychic anxiety (HAMD#10) with treatment with either minocycline or placebo. Numbers indicate the actual change in score from baseline to 12 weeks. Blue indicates an increase in symptom score, while red signifies reduction in score. HAMD, Hamilton Depression Rating Scale.

Of the 146 participants in the sample with high plasma CRP, week 12 data for the above-mentioned HAMD items were missing from 13 participants, leaving a total of *n* = 133 participants in the treatment response analysis. Of these, 117 received adjunctive simvastatin or placebo, and 16 received adjunctive minocycline or placebo. The results of regression analysis are presented in Table 3. There were three levels to the symptom score for the variables insomnia early in the night (HAMD item#4) and early morning insomnia (HAMD item#6) (i.e. these symptoms could be scored either 0, 1 or 2), thus these variables were assessed using ordinal regression. In our pooled data-set, psychic anxiety (HAMD item #10) had two levels (anxiety scores were either 0 or 1 for every participant in the data-set) and was assessed using logistic regression. Response to treatment with adjunctive simvastatin did not differ significantly from placebo added to standard treatment for any depression symptoms analysed. With a small sample size of 16 participants in the minocycline/placebo group, we were underpowered to perform regression analysis for adjunctive minocycline treatment. Therefore, the number and proportion of people in each treatment group whose symptom scores improved, did not change or worsened are presented in Table 4. Fisher's exact test was used to compare differences in the proportion of individuals whose symptoms did and did not improve (those whose symptoms worsened or did not differ from baseline) between minocycline and placebo groups. Overall, a higher proportion of people who received adjunctive minocycline experienced improvements in all three symptoms relative to those who received placebo plus standard treatment. This was only significant for anxiety (*P* = 0.003), as all participants who received minocycline reported improvements, compared with just 22% in the placebo group. A heat map visualising the degree of change in symptoms scores post-treatment with minocycline compared to placebo shows greater reductions in symptom scores with minocycline (Fig. 2).

**Table 3:** Response to simvastatin treatment for inflammation-associated symptoms in regression analysis

Symptom - HAMD item #	Odds ratio	95% CI		*P*
		Lower	Upper	
Insomnia (early) – 4	0.76	0.36	1.57	0.454^a^
Insomnia (morning) – 6	1.40	0.63	3.16	0.419^a^
Pyschic anxiety – 10	0.71	0.17	2.71	0.621^b^
Gastrointestinal – 12	1.20	0.70	2.09	0.505^a^
General somatic – 13	1.40	0.84	2.13	0.207^a^

HAMD, Hamilton Depression Rating Scale.

Statistical tests:

a. Ordinal regression.

b. Logistic regression.

**Table 4:** Response to treatment with minocycline

Symptom - HAMD item #	Treatment group *n* (%)		*P*
	Minocycline (*n* = 7)	Placebo (*n* = 9)	
**Insomnia (early) – 4**			0.060
Improved	6 (86%)	3 (33%)	
No change	1 (14%)	4 (44%)	
Worsened	0 (0%)	2 (22%)	
**Insomnia (waking) – 6**			0.315
Improved	5 (71%)	3 (33%)	
No change	1 (14%)	3 (33%)	
Worsened	1 (14%)	3 (33%)	
**Psychic anxiety – 10**			0.003
Improved	7 (100%)	2 (22%)	
No change	0 (0%)	2 (22%)	
Worsened	0 (0%)	5 (56%)	

HAMD, Hamilton Depression Rating Scale.

Statistical tests: Fisher's exact test.

## Discussion

The prevalence of inflammation in this LMIC sample with MDD was much higher (87%, *n* = 146) than the approximately 25% prevalence that has been reported from studies investigating associations between low-grade inflammation and depression in higher income countries.^28^ We previously found similarly high rates of low-grade inflammation in adult people with bipolar depression in Pakistan.^21^ Furthermore, there were a relatively high number of individuals in the sample with CRP values over 10 mg/L (*n* = 33). LMIC populations have heightened exposure to pro-inflammatory stressors like air pollution,^29^ pathogenic microbes^30^ and lower SES. This may have contributed to the high prevalence of inflammation seen in this sample of people with TRD, and may reflect higher levels of inflammation in Pakistan in general. Though it remains uncertain whether high rates of inflammation are unique to clinically depressed populations in this region, psychosocial and environmental stressors are known to increase the risk of MDD and predict poorer prognosis.^31^ It is possible that inflammatory processes may underlie this enhanced risk. Future research should aim to clarify the relationship between elevated inflammatory markers and depression by investigating rates of inflammation in depressed and non-clinical populations in Pakistan and other LMICs.

Inflammation has been shown to induce ‘sickness behaviours’ that mimic symptoms of MDD such as anhedonia, weight and appetite disturbances, fatigue and psychomotor slowing.^6^ Inflammation has also been associated with cognitive dysfunction, anxiety^6^ and suicidal ideation.^12^ Our analysis found early night and early morning insomnia and anxiety remained associated with elevated CRP when controlling for age, BMI, SES and gender. Associations between insomnia symptoms and inflammation are consistent with previous studies. Insomnia has been repeatedly associated with elevated CRP through large longitudinal and experimental studies.^6^ Furthermore, both sleep disturbance and inflammation have been shown to be influence the risk of depression.^32^ The association between elevated CRP and anxiety symptoms in depression is also well established.^6^ It should be noted, however, that this association only just met cut-offs for significance in our analysis, with a wide 95% CI indicating low precision (95% CI 1.39–21.7, *P* = 0.044). These results should therefore be interpreted cautiously, and further research should be conducted to confirm this association in LMIC settings.

Contrary to what has been reported previously,^6^ non-inflamed people in this sample were at greater risk of gastrointestinal and general somatic symptoms. Our group has previously reported insomnia in the middle of the night and suicidality to be negatively associated with low-grade inflammation in Pakistani adults with bipolar depression,^21^ which also contrasts with findings from higher income countries. These results indicate that depressive disorders may present differently in higher and lower income countries, and may reflect the unique contributing factors and vulnerabilities faced by the populations in each setting. Future research should aim to investigate the basis for these distinct associations in Pakistan and other LMICs.

When people with CRP levels of >10 mg/L were omitted, our linear regression analysis found that in addition to anxiety and insomnia, other symptoms positively associated with CRP included psychomotor retardation, agitation, and difficulties with work and other activities. These findings are largely consistent with the existing literature^6^ as well as sickness behaviour theory.^32^ Agitation has been associated with neuroinflammation in mental disorders,^34^ and increased agitation with higher baseline CRP may be a consequence of heightened anxiety and disturbed sleep. Symptoms negatively associated with CRP in linear regression were general somatic symptoms and weight loss. CRP has previously been associated with increased appetite in depressed populations,^35^ which may explain the negative association with weight loss observed here. It should be noted, however, that the original trials used different versions of the SPINREACT CRP-turbilatex agglutination test with differing sensitivities (the assay used in the simvastatin study had a lower detection limit of 1 mg/mL,^36^ while the lower limit in the minocycline study was 2 mg/L^37^). This limits any interpretation of linear regression results. These findings require replication in larger and more diverse LMIC patient samples, ideally using a higher sensitivity CRP assay.

Treatment with simvastatin did not improve insomnia or anxiety symptoms in Pakistani adults with TRD and high CRP. This finding is consistent with results from the original trial, which concluded simvastatin did not provide any therapeutic benefit over that of placebo overall in Pakistani adults with TRD.^15^ Moreover, a recent systematic review found no improvement in sleep-related symptoms with statin treatment in any study, and little to no improvement in anxiety.^38^ It is possible that the strength of the anti-inflammatory effects of simvastatin at the 20 mg dosage used in the parent RCT may not be adequate for the treatment of symptoms associated with inflammation.

In a smaller sample of 16 people, greater reductions in symptom scores were observed with adjunctive minocycline relative to placebo, particularly for anxiety symptoms, and to a lesser extent, early morning waking and early night insomnia. Despite the small sample size, our results are in line with other studies investigating minocycline augmentation treatment for MDD and TRD. In a pooled data analysis of 112 individuals with TRD which included people from the minocycline pilot trial included in the current analysis, Zazula et al^33^ reported that minocycline significantly improved anxiety symptom severity on the HAMD and the GAD-7, in addition to functional status and depressive symptoms.^39^ The use of minocycline to treat anxiety symptoms is also supported by a preclinical study which demonstrated chronic administration of minocycline reduced stress-induced anxiety behaviours.^39^ While larger and more diverse samples are needed, these results indicate augmentation with minocycline may be a viable strategy for treating comorbid depression and anxiety symptoms in people in Pakistan with elevated CRP, and possibly other LMIC settings.

This study was limited by a relatively small sample size, and by the availability of biomarker and other data measured in the original studies. MDD is a complex, heterogeneous condition, with many factors (e.g. genetics, adverse life experiences) contributing to symptom profiles that we were unable to assess in this analysis. Additionally, we did not correct for multiple comparisons. While correction for multiple comparisons was not necessary in this context given the exploratory nature of the analysis,^40^ our results would not withstand Bonferroni correction. Finally, our investigation of symptom response to minocycline was limited by a very small sample size, and so findings are difficult to extrapolate. Furthermore, as our analysis was exploratory, we did not compare the efficacy of each medication treatment directly. Therefore, at present, clinical inferences cannot be drawn from these results. Despite these limitations, this study provides preliminary evidence to support minocycline as an adjunctive treatment for a subset of inflammation-associated symptoms. Larger, more comprehensive trials investigating treatment response to minocycline for individual symptoms of depression may be warranted to confirm this finding.

Overall, this study's findings reveal that deeper insights about associations between inflammation and depression can be gained through investigating symptoms individually. Furthermore, these results support the notion that individuals with specific clinical phenotypes and evidence of peripheral inflammation may benefit from a more targeted and individualised approach with repurposed anti-inflammatory agents. Given the high prevalence of inflammation in this LMIC sample, targeting inflammation and increasing stress resilience may improve treatment outcomes for individuals with MDD in these settings.

## Supporting information

Fellows et al. supplementary material 1 Fellows et al. supplementary material

Fellows et al. supplementary material 2 Fellows et al. supplementary material

Fellows et al. supplementary material 3 Fellows et al. supplementary material

Fellows et al. supplementary material 4 Fellows et al. supplementary material

Fellows et al. supplementary material 5 Fellows et al. supplementary material

## Data Availability

Data will be made available upon request to the corresponding author.

## References

[1] Chand SP, Arif H. Depression. In StatPearls. StatPearls Publishing, 2024 (https://www.ncbi.nlm.nih.gov/books/NBK430847/). 28613597

[2] Friedrich M. Depression is the leading cause of disability around the world. JAMA 2017; 317(15): 1517. 10.1001/jama.2017.3826 28418490

[3] World Health Organization. Depressive Disorder (Depression). World Health Organization, 2023 (https://www.who.int/news-room/fact-sheets/detail/depression).

[4] Akil H, Gordon J, Hen R, Javitch J, Mayberg H, McEwen B, et al. Treatment resistant depression: a multi-scale, systems biology approach. Neurosci Biobehav Rev 2018; 84: 272–88. 28859997 10.1016/j.neubiorev.2017.08.019 PMC5729118

[5] Yoshimura R, Hori H, Ikenouchi-Sugita A, Umene-Nakano W, Ueda N, Nakamura J. Higher plasma interleukin-6 (IL-6) level is associated with SSRI- or SNRI-refractory depression. Prog Neuropsychopharmacol Biol Psychiatry 2009; 33(4): 722–6. 19332097 10.1016/j.pnpbp.2009.03.020

[6] Orsolini L, Pompili S, Tempia Valenta S, Salvi V, Volpe U. C-Reactive protein as a biomarker for major depressive disorder? Int J Mol Sci 2022; 23(3): 1616. 35163538 10.3390/ijms23031616 PMC8836046

[7] Qiao J, Geng D, Qian L, Zhu X, Zhao H. Correlation of clinical features with hs-CRP in TRD patients. Exp Ther Med 2019; 17(1): 344–8. 30651801 10.3892/etm.2018.6914 PMC6307477

[8] Bermudez EA, Rifai M, Burning J, Manson JE, Ridker PM. Interrelationships among circulating interleukin-6, C-reactive protein, and traditional cardiovascular risk factors in women. Arterioscler Thromb Vasc Biol 2002; 22: 1668–73. 12377747 10.1161/01.atv.0000029781.31325.66

[9] Patel S, Keating BA, Dale RC. Anti-inflammatory properties of commonly used psychiatric drugs. Front Neurosci 2023; 16: 1039379. 36704001 10.3389/fnins.2022.1039379 PMC9871790

[10] Husain MI, Strawbridge R, Stokes PR, Young AH. Anti-inflammatory treatments for mood disorders: systematic review and meta-analysis. J Psychopharmacol 2017; 31(9): 1137–48. 28858537 10.1177/0269881117725711

[11] Simon MS, Arteaga-Henríquez G, Fouad Algendy A, Siepmann T, Illigens BMW. Anti-inflammatory treatment efficacy in major depressive disorder: a systematic review of meta-analyses. Neuropsychiatr Dis Treat 2023; 19: 1–25. 36636142 10.2147/NDT.S385117 PMC9830720

[12] Miola A, Dal Porto V, Tadmor T, Croatto G, Scocco P, Manchia M, et al. Increased C-reactive protein concentration and suicidal behavior in people with psychiatric disorders: a systematic review and meta-analysis. Acta Psychiatr Scand 2021; 144(6): 537–52. 34292580 10.1111/acps.13351 PMC9290832

[13] World Bank, Population, Total for Lower Middle Income Countries [SPPOPTOTLLMC]. FRED, Federal Reserve Bank of St. Louis. 2023 (https://fred.stlouisfed.org/series/SPPOPTOTLLMC).

[14] Rathod S, Pinninti N, Irfan M, Gorczynski P, Rathod P, Gega L, et al. Mental health service provision in low- and middle-income countries. Health Serv Insights 2017; 10: 1178632917694350. 28469456 10.1177/1178632917694350 PMC5398308

[15] Husain MI, Chaudhry IB, Khoso AB, Kiran T, Khan N, Ahmad F, et al. Effect of adjunctive simvastatin on depressive symptoms among adults with treatment-resistant depression: a randomized clinical trial. JAMA Netw Open 2023; 6(2): e230147. 36808239 10.1001/jamanetworkopen.2023.0147 PMC9941891

[16] Husain MI, Chaudhry IB, Husain N, Khoso AB, Rahman RR, Hamirani MM, et al. Minocycline as an adjunct for treatment-resistant depressive symptoms: a pilot randomised placebo-controlled trial. J Psychopharmacol 2017; 31(9): 1166–75. 28857658 10.1177/0269881117724352

[17] Dessoki HH, Khattab RAER, Moris W, Abdelhakim AAE, Lotfy AMM, Salah H. C-reactive protein as a biomarker for unipolar versus bipolar depression: a cross-sectional study. Middle East Curr Psychiatry 2023; 30: 69.

[18] Ridker PM. Clinical application of C-reactive protein for cardiovascular disease detection and prevention. Circulation 2003; 107(3): 363–9. 12551853 10.1161/01.cir.0000053730.47739.3c

[19] Chamberlain SR, Cavanagh J, de Boer P, Mondelli V, Jones DNC, Drevets WC, et al. Treatment-resistant depression and peripheral C-reactive protein. Br J Psychiatry 2019; 214(1): 11–9. 29764522 10.1192/bjp.2018.66 PMC6124647

[20] Felger JC, Haroon E, Patel TA, Goldsmith DR, Wommack EC, Woolwine BJ, et al. What does plasma CRP tell us about peripheral and central inflammation in depression? Mol Psychiatry 2020; 25: 1301–11. 29895893 10.1038/s41380-018-0096-3 PMC6291384

[21] Jones B, Mahmood U, Hodsoll J, Chaudhry I, Khoso A, Husain M. Associations between peripheral inflammation and clinical phenotypes of bipolar depression in a lower-middle income country. CNS Spectr 2023; 28(6): 710–8. 37160707 10.1017/S1092852923002316

[22] Hamilton M. A rating scale for depression. J Neurol Neurosurg Psychiatry 1960; 23(1): 56–62. 14399272 10.1136/jnnp.23.1.56 PMC495331

[23] Spitzer RL, Kroenke K, Williams JBW, Löwe B. A brief measure for assessing generalized anxiety disorder: the GAD-7. Arch Intern Med 2006; 166(10): 1092–7. 16717171 10.1001/archinte.166.10.1092

[24] Busner J, Targum SD. The clinical global impressions scale: applying a research tool in clinical practice. Psychiatry (Edgmont) 2007; 4(7): 28–37. PMC2880930 20526405

[25] Kroenke K, Spitzer RL, Williams JB. The PHQ-9: validity of a brief depression severity measure. J Gen Intern Med 2001; 16(9): 606–13. 11556941 10.1046/j.1525-1497.2001.016009606.x PMC1495268

[26] R Core Team. R: A Language and Environment for Statistical Computing. R Foundation for Statistical Computing, 2023 (https://www.R-project.org).

[27] Nehring SM, Goyal A, Patel BC. C reactive protein. In StatPearls. StatPearls Publishing, 2024 (https://www.ncbi.nlm.nih.gov/books/NBK441843/). 28722873

[28] Osimo EF, Baxter LJ, Lewis G, Jones PB, Khandaker GM. Prevalence of low-grade inflammation in depression: a systematic review and meta-analysis of CRP levels. Psychol Med 2019; 49(12): 1958–70. 31258105 10.1017/S0033291719001454 PMC6712955

[29] Arias-Pérez RD, Taborda NA, Gómez DM, Narvaez JF, Porras J, Hernandez JC. Inflammatory effects of particulate matter air pollution. Environ Sci Pollut Res Int 2020; 27(34): 42390–404. 32870429 10.1007/s11356-020-10574-w

[30] Goddard FGB, Ban R, Barr DB, Brown J, Cannon J, Colford JM Jr, et al. Measuring environmental exposure to enteric pathogens in low-income settings: review and recommendations of an interdisciplinary working group. Environ Sci Technol 2020; 54(19): 11673–91. 32813503 10.1021/acs.est.0c02421 PMC7547864

[31] Gilman SE, Trinh NH, Smoller JW, Fava M, Murphy JM, Breslau J. Psychosocial stressors and the prognosis of major depression: a test of axis IV. Psychol Med 2013; 43(2): 303–16. 22640506 10.1017/S0033291712001080 PMC3721739

[32] Irwin MR, Olmstead R, Carroll JE. Sleep disturbance, sleep duration, and inflammation: a systematic review and meta-analysis of cohort studies and experimental sleep deprivation. Biol Psychiatry 2016; 80(1): 40–52. 26140821 10.1016/j.biopsych.2015.05.014 PMC4666828

[33] Zazula R, Husain MI, Mohebbi M, Walker AJ, Chaudhry IB, Khoso AB. Minocycline as adjunctive treatment for major depressive disorder: pooled data from two randomized controlled trials. Aust N Z J Psychiatry 2021; 55(8): 784–98. 33092404 10.1177/0004867420965697

[34] Larsen JB, Stunes AK, Vaaler A, Reitan SK. Cytokines in agitated and non-agitated patients admitted to an acute psychiatric department: a cross-sectional study. PLoS One 2019; 14(9): e0222242. 31509578 10.1371/journal.pone.0222242 PMC6738632

[35] Chae WR, Baumert J, Nübel J, Brasanac J, Gold SM, Hapke U, et al. Associations between individual depressive symptoms and immunometabolic characteristics in major depression. Eur Neuropsychopharmacol 2023; 71: 25–40. 36966710 10.1016/j.euroneuro.2023.03.007

[36] SPINREACT. CRP-turbilatex Product Insert. 2021 (https://www.spinreact.com/assets/files/Inserts/MX-BS800/mxtlis40_crp-4-1-2021.pdf).

[37] SPINREACT. CRP-turbilatex Product Insert. 2017 (https://www.spinreact.com/files/Inserts/MD/TURBILATEX/MDTLIS40_CRP__4+1_2017.pdf).

[38] De Giorgi R, Rizzo Pesci N, Quinton A, De Crescenzo F, Cowen PJ, Harmer CJ. Statins in depression: an evidence-based overview of mechanisms and clinical studies. Front Psychiatry 2021; 12: 702617. 34385939 10.3389/fpsyt.2021.702617 PMC8353114

[39] Liu HY, Yue J, Hu LN, Cheng LF, Wang XS, Wang XJ, et al. Chronic minocycline treatment reduces the anxiety-like behaviors induced by repeated restraint stress through modulating neuroinflammation. Brain Res Bull 2018; 143: 19–26. 30149196 10.1016/j.brainresbull.2018.08.015

[40] Bender R, Lange S. Adjusting for multiple-testing—when and how? J Clin Epidemiol 2001; 54(4): 343–9. 11297884 10.1016/s0895-4356(00)00314-0

