# Supplementary figures and images for: Associations between C-reactive protein and individual symptoms of depression in a lower-middle income country
Source: BJPsych Open. 2024 Oct 3;10(5):e169. doi: 10.1192/bjo.2024.735 (PMC11536211; doi:10.1192/bjo.2024.735)

**HAMD Item #4**

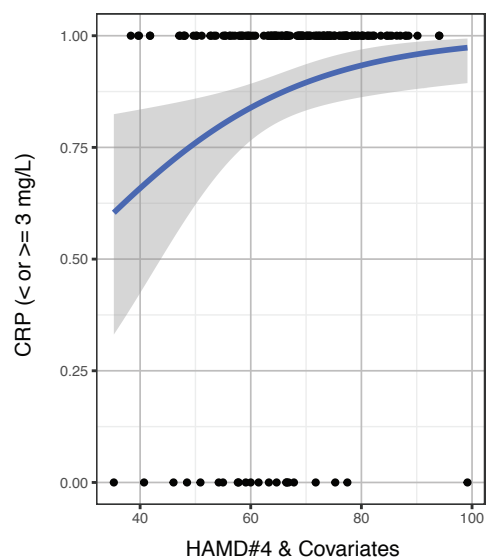

**HAMD Item #6**

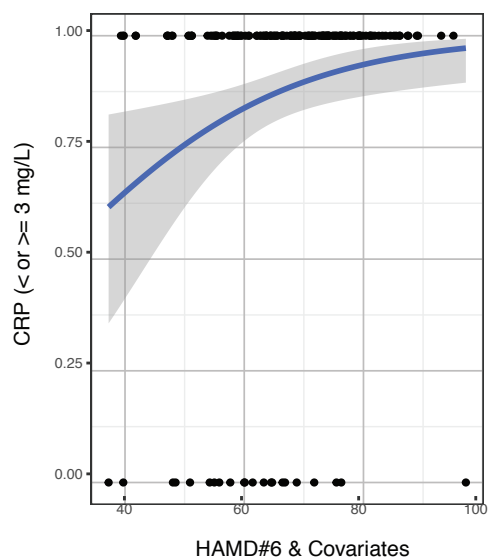

**HAMD Item #10**

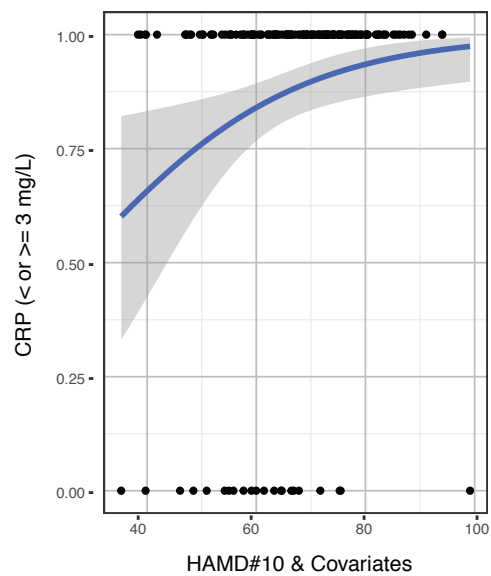

**HAMD Item #12**

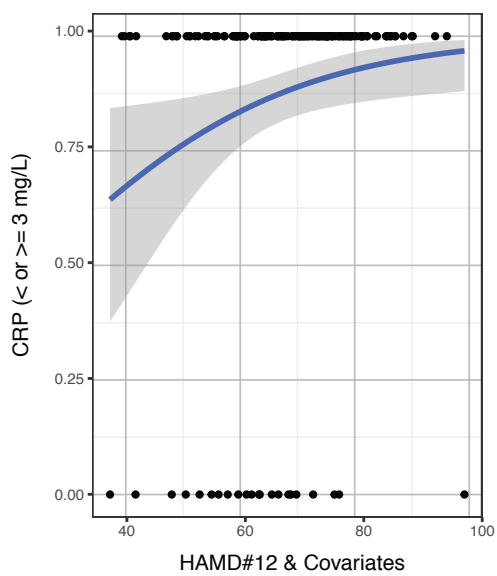

**HAMD Item #13**

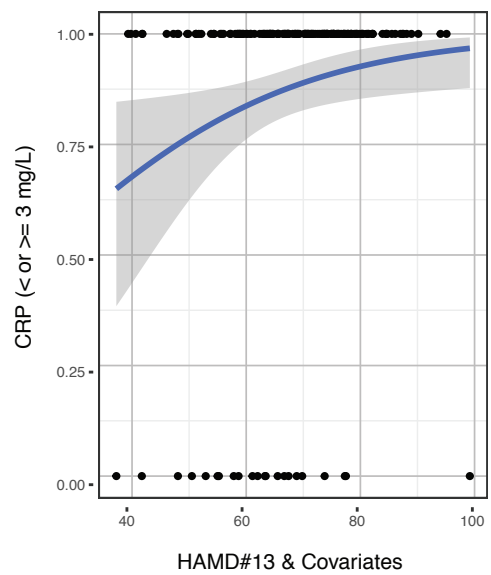

Supplement: Fellows et al. supplementary material 1 — Fellows et al. supplementary material [file S205647242400735Xsup001.pdf]

**HAMD Item #4**

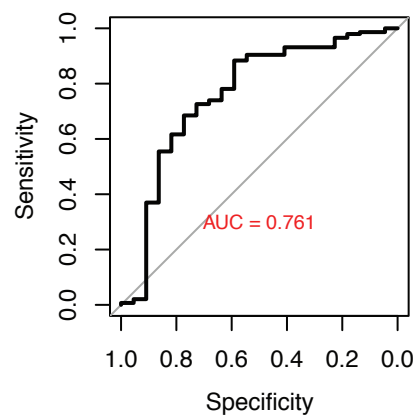

**HAMD Item #6**

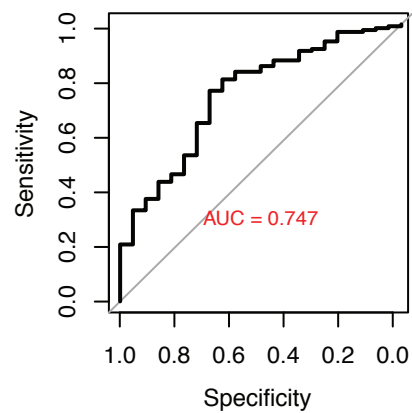

**HAMD Item #10**

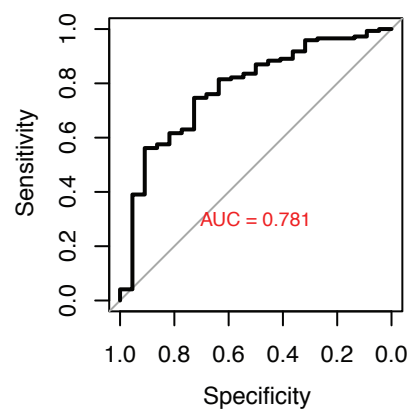

**HAMD Item #12**

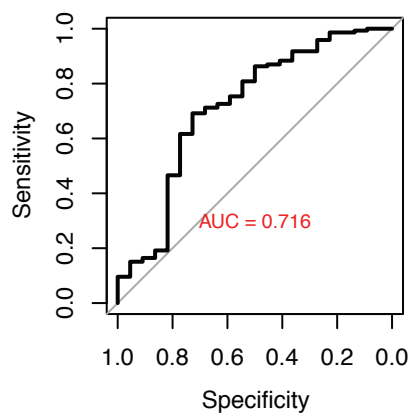

**HAMD Item #13**

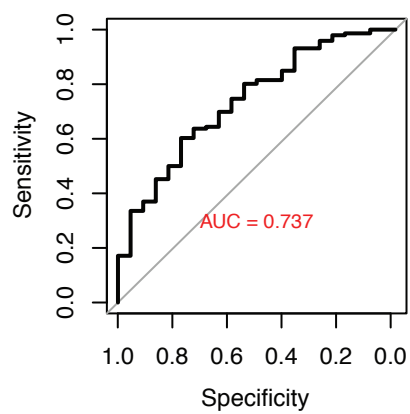

Supplement: Fellows et al. supplementary material 2 — Fellows et al. supplementary material [file S205647242400735Xsup002.pdf]
